# Role for astroglial α1-adrenoreceptors in gliotransmission and control of synaptic plasticity in the neocortex

**DOI:** 10.3389/fncel.2015.00230

**Published:** 2015-06-16

**Authors:** Yuriy Pankratov, Ulyana Lalo

**Affiliations:** School of Life Sciences, Gibbet Hill Campus, University of WarwickCoventry, UK

**Keywords:** astrocyte-neuron interactions, exocytosis, norepinephrine, ATP release, P2X receptor, desensitization, metaplasticy, D-serine

## Abstract

Communication between neuronal and glial cells is thought to be very important for many brain functions. Acting via release of gliotransmitters, astrocytes can modulate synaptic strength. The mechanisms underlying gliotransmission remain uncertain with exocytosis being the most intriguing and debated pathway. We demonstrate that astroglial α1-adrenoreceptors are very sensitive to noradrenaline (NA) and make a significant contribution to intracellular Ca^2+^-signaling in layer 2/3 neocortical astrocytes. We also show that astroglial α1-adrenoreceptors are prone to desensitization upon prolonged exposure to NA. We show that within neocortical slices, α-1adrenoreceptors can activate vesicular release of ATP and D-serine from cortical astrocytes which initiate a burst of ATP receptor-mediated currents in adjacent pyramidal neurons. These purinergic currents can be inhibited by intracellular perfusion of astrocytes with Tetanus Toxin light chain, verifying their origin via astroglial exocytosis. We show that α1 adrenoreceptor-activated release of gliotransmitters is important for the induction of synaptic plasticity in the neocortex:long-term potentiation (LTP) of neocortical excitatory synaptic potentials can be abolished by the selective α1-adrenoreceptor antagonist terazosin. We show that weak sub-threshold theta-burst stimulation (TBS) can induce LTP when astrocytes are additionally activated by 1 μM NA. This facilitation is dependent on the activation of neuronal ATP receptors and is abolished in neocortical slices from dn-SNARE mice which have impaired glial exocytosis. Importantly, facilitation of LTP by NA can be significantly reduced by perfusion of individual astrocytes with Tetanus Toxin. Our results strongly support the physiological importance of astroglial adrenergic signaling and exocytosis of gliotransmitters for modulation of synaptic transmission and plasticity.

## Introduction

It is generally acknowledged that astrocytes are very important components of brain networks. The astroglial network receives and integrates signals from neurons and responds by increasing the metabolic support for neurons and by modulating neuronal activity via the release of gliotransmitters (Giaume et al., 2010; Halassa and Haydon, 2010). The variety of physiological functions of astroglia spans from “housekeeping”, such as clearance and turnover of neurotransmittes and ionic homeostasis to the control of neuro-vascular coupling and modulation of synaptic transmission and plasticity (Gordon et al., 2009; Attwell et al., 2010; Giaume et al., 2010; Henneberger et al., 2010; Min and Nevian, 2012). It is commonly accepted that elevation of cytosolic calcium is the major mechanism of astrocyte activation and integration of information within the glial network and underpins many functions of astrocytes.

Astrocytes are endowed with an extensive complement of Gq protein-coupled neurotransmitter receptors that trigger IP3-mediated release of Ca^2+^ from intracellular stores (Halassa and Haydon, 2010; Araque et al., 2014; Khakh and McCarthy, 2015). In addition, ionotropic Ca^2+^-permeable receptors can contribute to astroglial signaling (Palygin et al., 2011). There is an accumulating evidence that Ca^2+^signaling in brain astrocytes can be activated by synaptically-derived neurotransmitters (Lalo et al., 2011a, 2014a; Palygin et al., 2011; Panatier et al., 2011), by the local autocrine release of gliotransmitters such as glutamate or ATP (Araque et al., 2014) and via diffuse volume-transmitted neuromodulators such as serotonin, acetylcholine or noradrenaline (NA; Schipke et al., 2008; Ding et al., 2013; Paukert et al., 2014; Khakh and McCarthy, 2015). Although the importance of all modes of astroglial signaling is generally recognized, their specific physiological roles are yet to be fully understood (Araque et al., 2014; Khakh and McCarthy, 2015). It was shown that receptors to serotonin and acetylcholine do not bring significant contribution to calcium dynamics in cortical astrocytes (Schipke et al., 2008) whereas astrocytic α1-adrenoreceptros (α1-ARs) were recently highlighted as important participants in calcium signaling (Ding et al., 2013; Paukert et al., 2014).

Astroglial Ca^2+^-signaling is tightly linked to the release of gliotransmitters which plays an important role in glia-neuron communication (Halassa et al., 2007; Araque et al., 2014). It has been widely reported that the release of ATP (Lalo et al., 2014a, b), glutamate and D-serine from astrocytes can modulate the activity of neuronal excitatory and inhibitory synapses (Gordon et al., 2009; Panatier et al., 2011; Lalo et al., 2014a), long-term synaptic plasticity (Pascual et al., 2005; Henneberger et al., 2010; Araque et al., 2014; Lalo et al., 2014b; Rasooli-Nejad et al., 2014) and neurovascular coupling (Attwell et al., 2010; Gourine et al., 2010). Although detailed mechanisms of gliotransmission remain uncertain, the importance of Ca^2+^-dependent vesicular and non-vesicular pathways has been recently reported (Gourine et al., 2010; Woo et al., 2012; Araque et al., 2014; Lalo et al., 2014a).

Thus, it is conceivable that astroglial α1-ARs may be involved in glial control of synaptic activity. A number of recent studies have used NA as a tool for the activation of astrocytes and showed that NA-evoked and glia-derived ATP can modulate excitatory synapses in the hippocampus and hypothalamus (Gordon et al., 2009; Pougnet et al., 2014). However these papers do not provide direct evidence for adrenoreceptor-triggered release of gliotransmitters and report opposing effects of glia-derived ATP on AMPA receptors trafficking. As such, the functional properties of noradrenergic glial signaling and its contribution to astroglial control of synaptic plasticity require further investigation. In the present study, we demonstrate that astroglial α1-ARs can activate exocytosis of gliotransmitters, in particular ATP, and this mechanism contributes to modulation of synaptic plasticity in neocortical neurons. To verify this, we use a combination of approaches including transgenic mice with inducible astroglial expression of dominant-negative SNARE domain (dn-SNARE; Pascual et al., 2005), intracellular perfusion of astrocytes with Ca^2+^-chelators and inhibitors of SNARE proteins.

## Material and Methods

All animal work has been carried out in accordance with UK legislation and “3R” strategy; research has not involved non-human primates.

Experiments were performed on astrocytes and neurons of somato-sensory cortex of dn-SNARE transgenic mice (Pascual et al., 2005; Halassa et al., 2009), their wild-type littermates (WT) and transgenic mice expressing enhanced green fluorescent protein (EGFP) under the control of the glial fibrillary acidic protein (GFAP) promoter (Lalo et al., 2011a; Palygin et al., 2011). Data obtained in the experiments on GFAP-EGFP (GFEC) mice did not differ significantly from data obtained in the WT mice. For clarity, all data referred to here as WT are reported solely for WT littermates to dn-SNARE mice; usage of GFAP-EGFP mice was explicitly stated where appropriate.

### Slice and Cell Preparation

Mice (8–12 weeks) were anesthetized by halothane and then decapitated, in accordance with UK legislation. Brains were removed rapidly after decapitation and placed into ice-cold physiological saline containing (mM): NaCl 130, KCl_3_, CaCl_2_ 0.5, MgCl_2_ 2.5, NaH_2_PO_4_ 1, NaHCO_3_ 25, glucose 15, pH of 7.4 gassed with 95% O_2_—5% CO_2_. Transverse slices (280 μm) were cut at 4°C and then placed in physiological saline containing (mM): NaCl 130, KCl_3_, CaCl_2_ 2.5, MgCl_2_ 1, NaH_2_PO_4_ 1, NaHCO_3_ 22, glucose 15, pH of 7.4 gassed with 95% O_2_—5% CO_2_ and kept for 1–4 h prior to cell isolation and recording. Neocortical pyramidal neurons were acutely isolated using the modified “vibrating” technique (Lalo et al., 2011a; Rasooli-Nejad et al., 2014). The glass ball (200 μm diameter) was moved slowly some 10–50 μm above the slice surface, while vibrating at 100 Hz (lateral displacements 20–30 μm). This technique mechanically isolates cells whilst preserving the function of membrane proteins and is therefore devoid of many artifacts of enzymatic cell isolation and culturing procedures. The composition of extracellular solution for all isolated cell experiments was (mM): 135 NaCl; 2.7 KCl; 2.5 CaCl_2_; 1 MgCl_2_; 10 HEPES, 1 NaH_2_PO_4,_ 15 glucose, pH adjusted with NaOH to 7.3.

Astrocytes were identified by their morphology under DIC observation, EGFP fluorescence (astrocytes from dn-SNARE and GFAP-EGFP mice) or staining with sulforhodamine 101 (astrocytes from WT mice). After recording, the identification of astrocyte was confirmed via functional properties (high potassium conductance, low input resistance, strong activity of glutamate transporters) as described previously (Lalo et al., 2011a; Palygin et al., 2011).

### Electrophysiological Recordings

Whole-cell voltage clamp recordings from neocortical neurons and astrocytes were made with patch pipettes (4–5 MΩ for neurons and 6–8 MΩ for astrocytes) filled with intracellular solution (in mM): 110 KCl, 10 NaCl, 10 HEPES, 5 MgATP, 10 EGTA, 1 CaCl_2_, pH 7.35. Currents were monitored using an AxoPatch200B patch-clamp amplifier (Axon Instruments, USA) filtered at 2 kHz and digitized at 4 kHz. Experiments were controlled by PCI-6229 data acquisition board (NI, USA) and WinFluor software (Strathclyde University, UK); data were analyzed with custom software. Liquid junction potentials were compensated with the patch-clamp amplifier. Series and input resistances were respectively 5–7 MΩ and 500–1100 MΩ in neurons and 8–12 MΩ and 50–150 MΩ in astrocytes; both series and input resistance varied by less than 20% in the cells accepted for analysis. For activation of synaptic inputs, axons originating from layer IV–VI neurons were stimulated with a bipolar coaxial electrode (WPI, USA) placed in layer V close to the layer IV border, approximately opposite the site of recording; stimulus duration was 300 μs. The stimulus magnitude was set 3–4 times higher than the minimal stimulus necessary to elicit a response in layer II pyramidal neurons (Lalo et al., 2011a; Palygin et al., 2011; Lalo et al., 2014a).

Field excitatory postsynaptic potentials (fEPSPs) were measured via a glass micropipette filled with extracellular solution (0.5–1 MΩ resistance) placed in neocortical layer II/III. In order to induce long-term plasticity of EPSPs two or five episode of theta-burst stimulation (HFS) were delivered; each HFS episode consisted of five pulses of 100 Hz stimulation, repeated 10 times with 200 ms interval (total 50 pulses per episode).

### Multi-photon Fluorescent Ca^2+^-imaging in astrocytes

To monitor the cytoplasmic free Ca^2+^concentraton ([Ca^2+^]_i_) *in situ*, astrocytes of neocortical slices were loaded via 30 min incubation with 1 μM of Rhod-2AM or Calcium Green-2AM and sulphorhodamine 101 (WT mice) at 33°C. Two-photon imaging of neurons and astrocytes was performed using a Zeiss LSM-7MP multi-photon microscope coupled to a SpectraPhysics MaiTai pulsing laser; experiments were controlled by ZEN LSM software (Carl Zeiss, Germany). Images were further analyzed off-line using ZEN LSM (Carl Zeiss) and ImageJ (NIH) software. The [Ca^2+^]_i_ levels were expressed as ΔF/F ratio averaged over a region of interest (ROI). For analysis of spontaneous Ca^2+^–transients in astrocytes, three ROIs located over dendrites and one ROI located over the soma were chosen. Overall Ca^2+^-response to adrenoreceptor agonists or synaptic stimulation was quantified using an ROI covering the whole cell image.

The NA and other drugs were applied to the recording chamber using gravity-feed application system with the flow rate of 5 mL/min. The recording chamber volume was 1.2 mL, so the time of full solution exchange could be estimated as 20–30 s. In measurements of the concentration-dependent response to adrenoreceptors agonists, only two or three applications of agonist were made to each neocortical slice in order to minimize the potential effect of receptor desensitization. The reference concentration of agonist (10 μM for NA, 300 nM for A61603) was used in all experiments; the lowest concentrations were applied first. Amplitudes of all responses were normalized to the response to the reference concentration. Data were then pooled together and fitted with a sigmoidal relationship to obtain the EC_50_ and Hill coefficient. Data on the concentration-dependence for the adrenoreceptors agonists were fitted with the following equation: ${\text{F/F}}_{\text{ref}}\text{ = }{\left(\left[\text{A}\right]{\text{/EC}}_{\text{50}}\right)}^{\text{p}}\text{/}\left(\text{1+}{\left(\left[\text{A}\right]{\text{/EC}}_{\text{50}}\right)}^{\text{p}}\right)$, where F is the amplitude of response to the agonist concentration [A], F_ref_ is the response to the reference concentration, p is the Hill coefficient.

### Measurement of Extracellular Concentration of ATP and D-serine in the Brain Tissue

The concentration of ATP within cortical slices was measured using microelectrode biosensors obtained from Sarissa Biomedical Ltd. (Coventry, UK). A detailed description of the properties of biosensors and recording procedure has been published previously (Frenguelli et al., 2007). Briefly, biosensors consisted of ATP or D-serine metabolizing enzymes immobilized within a matrix on thin (25 μM) Pt/Ir wire. This allowed insertion of the sensors into the cortical slice and minimized the influence of a layer of dead surface tissue. ATP and D-serine biosensors were used simultaneously. A third, null, biosensor was also used. This sensor is identical to the ATP and D-serine sensors and has a matrix, but lacks enzymes. The signal from the null sensor was subtracted from the signal obtained on the ATP and D-serine sensor. This allows the contribution of any non-specific electroactive substances that bypass the sensor screening layer to be eliminated. Biosensors show a linear response to increasing concentration of ATP and D-serine and have a rise time less than 10 s (Frenguelli et al., 2007). Biosensors were calibrated with a known concentrations (10 μM) of ATP and D-serine before the slice was present in the perfusion chamber and after the slice had been removed. This allowed compensation of any reduction in sensitivity during the experiment. The integrity of the screening layer was assessed with 10 μM 5-HT. Biosensor signals were acquired at 1 kHz with a 1400 CED interface and analyzed using Spike 6.1 software (Cambridge Electronics Design, Cambridge, UK).

### Data Analysis

All data are presented as mean ± SD and the statistical significance of differences between data groups was tested by two-tailed unpaired *t*-test, unless indicated otherwise. Each neocortical slice was used only for one experiment (e.g., fluorescent recordings in single astrocyte or single long-term potentiation (LTP) induction experiment). The number of experiments/cells reported is therefore equal to the number of slices used. The experimental protocols were allocated randomly so the data in any group were drawn from at least three animals.

The spontaneous transmembrane currents recorded in neurons were analyzed off-line using methods described previously (Pankratov et al., 2007; Lalo et al., 2011a; Palygin et al., 2011). The amplitude distributions of spontaneous and evoked currents were analyzed with the aid of probability density functions and likelihood maximization techniques; all histograms shown were calculated as probability density functions. The amplitude distributions were fitted with either multi-quantal binomial model or bi-modal function consisting of two Gaussians with variable peak location, width and amplitude. The decay time distributions were fitted with bi-modal functions. Parameters of models were fit using likelihood maximization routine. To monitor and analyze the time course of changes in the amplitude and frequency of spontaneous currents, the amplitude and frequency were averaged over a 1 min time window.

## Results

### Functional Properties of Adrenergic Ca^2+^ Signaling in Neocortical Astrocytes *In Situ*

**Figure 1:**
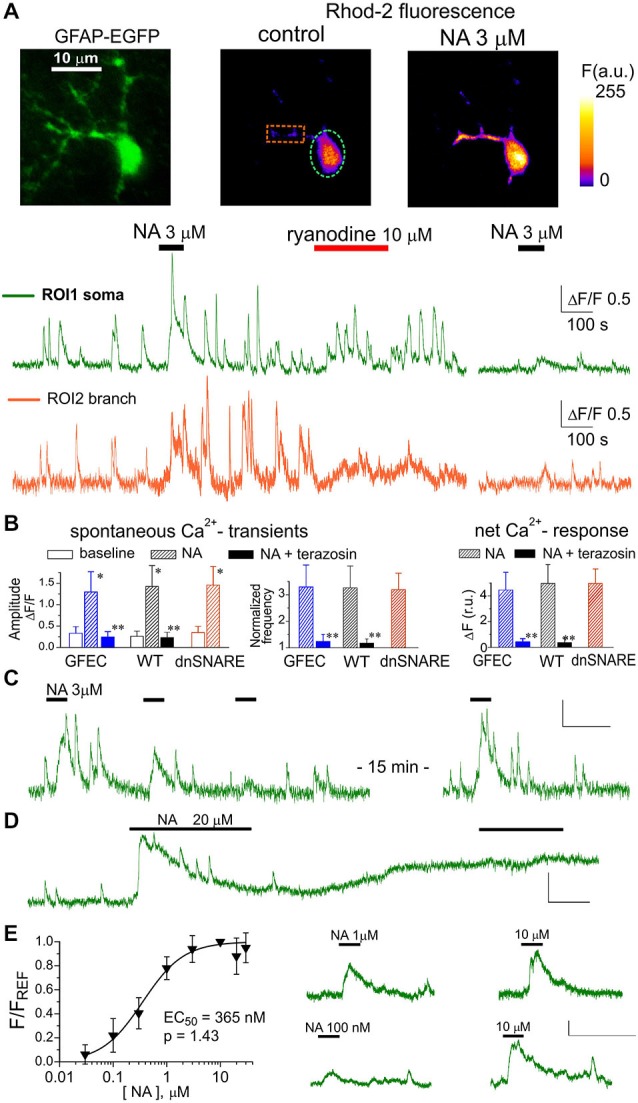
**α1-adrenoreceptors contribute to Ca^2+^ signaling in cortical astrocytes. (A)** Representative multi-photon images of enhanced green fluorescent protein (EGFP) fluorescence and presudo-color images of Rhod-2 fluorescence recorded in the astrocytes from glial fibrillary acidic protein (GFAP)-EGFP (GFEC) mouse before (control) and after the application of noradrenaline (NA). Graphs below show the time course of Rhod-2 fluorescence averaged over regions indicated in fluorescence images. Note the marked spontaneous elevations in the Ca^2+^ level, which were enhanced by application of NA. Note the significant NA-activated response in the astrocytic branch and inhibition of response to NA 10 min after application of ryanodine. **(B)** The pooled data on peak amplitude and frequency of spontaneous Ca^2+^-transients and the net response to the NA recorded in astrocytes of different mice strains in control and in the presence of α1-AR antagonist terazosin (30 nM). Frequency of spontaneous transients (middle graph) was measured within 3 min after application of NA and was normalized to baseline value. Net response was evaluated as an integral Ca^2+^-signal measured during 3 min after NA application, averaged over the whole cell image and normalized to the integral Ca^2+^ signal measured during 3 min before NA application. Data are shown as mean ± SD for seven cells in control (for each strain) and six cells under terazosin (WT and GFEC). In the each strain, asterisks (*) indicate statistical significance of effect of NA on the peak amplitude of Ca^2+^-transients in comparison to the corresponding baseline value, double asterisks (**) indicate significance of inhibitory effect of terazosin in comparison to the effect of NA alone; *P* < 0.01 given by *t*-test in both cases. **(C)** repetitive application of NA (3 μM) with 5 min interval causes the desensitization of the response; **(D)** prolonged application of 20 μM NA leads to the elevated Ca^2+^ level and non-responsiveness of neocortical astrocytes; **(E)** the concentration-dependence of net Ca^2+^-transients evoked by NA in cortical astrocytes was assessed as described in the *Methods*; each point show mean ± SD for 4–5 cells. Fluorescent signals shown in panels **(C–E)** were integrated over the cell somata; all scale bars are ΔF 0.5 and 200 s.

Under basal conditions (before application of NA), astrocytes in neocortical slices from all mice strains exhibited spontaneous Ca^2+^ transients with the average frequency varying in the range of 0.5–2.1 min^−1^ (Figure 1A). Bath application of 3 μM NA (60 s long) induced robust Ca^2+^ elevations both in the soma and branches and increased the amplitude and frequency of Ca^2+^ transients (Figures 1A,B). There was no statistically significant difference in the action of NA between astrocytes of different strains (Figure 1B). The action of NA was mimicked by the specific α1-AR agonist A61603 (Yoshiki et al., 2013) in all 13 cells tested (data not shown).

At the same time, astroglial α1-ARs showed evidence of desensitization. In all eight astrocytes tested (WT mice), repetitive application of NA (3 μM) or A61603 (100 nM) with intervals shorter than 5 min caused marked reduction in the response amplitude and lead to a period of non-responsiveness, typically lasting 10–15 min (Figure 1C). Moreover, application of NA in concentrations greater than 10 μM lead to a long-lasting period of elevated intracellular Ca^2+^ in 8 out of 10 cells tested. During this period, astrocytes did not respond further to NA and did not exhibit notable spontaneous activity (Figure 1D). Elevation in the baseline level of cytosolic Ca^2+^ reached 44 ± 17% (*n* = 8) and was statistically significant (*P* < 0.01, compared to the control level using paired *t*-test). The susceptibility of α1AR-mediated responses to desensitization suggests the existence of finely-tuned molecular mechanisms preventing their overstimulation, likely to avoid Ca^2+^-overload.

The predominant contribution of astroglial α1-ARs to responses evoked by NA was confirmed by the selective α1-AR antagonist terazosin. Application of terazosin (30 nM) effectively blocked responses to NA in all 12 astrocytes tested (Figure 1B). Terazosin also had a considerable inhibitory effect on spontaneous Ca^2+^-transients (Figure 1B). This suggests either a basal tone of NA in neocortical slices, or constitutive α1-AR activity.

Rather surprisingly, NA-evoked responses in cortical astrocytes were sensitive to ryanodine. Application of 10 μM ryanodine caused an initial augmentation of spontaneous Ca^2+^-signaling, mainly in the soma, which lasted typically for 5–10 min and was followed by an irreversible inhibition of spontaneous transients (Figure 1A). Such an action is typical for ryanodine modulation of calcium-induced calcium release (CICR) mechanism (Zucchi and Ronca-Testoni, 1997). When NA was applied after cessation of spontaneous signaling (10–15 min after ryanodine) it produced a much reduced Ca^2+^ response in all seven astrocytes tested (Figures 1A,B). These results suggest the significant role of ryanodine receptor-mediated CICR in the amplification of astroglial Ca^2+^ signaling.

The α1-ARs of neocortical astrocytes showed rather high sensitivity; they could be activated by NA in sub-micromolar range (Figure 1D). The EC_50_ for NA-activated net Ca^2+^ response was 365 ± 26 nM (*n* = 18, WT mice) with a Hill coefficient of 1.43. The EC_50_ for the specific α1-agonist A61603 was 18.9 ± 5.1 nM (*n* = 13) with a Hill coefficient of 1.45. Large values for Hill coefficients are, very likely, related to the amplification of the responses to higher concentrations by CICR mechanism.

**Figure 2:**
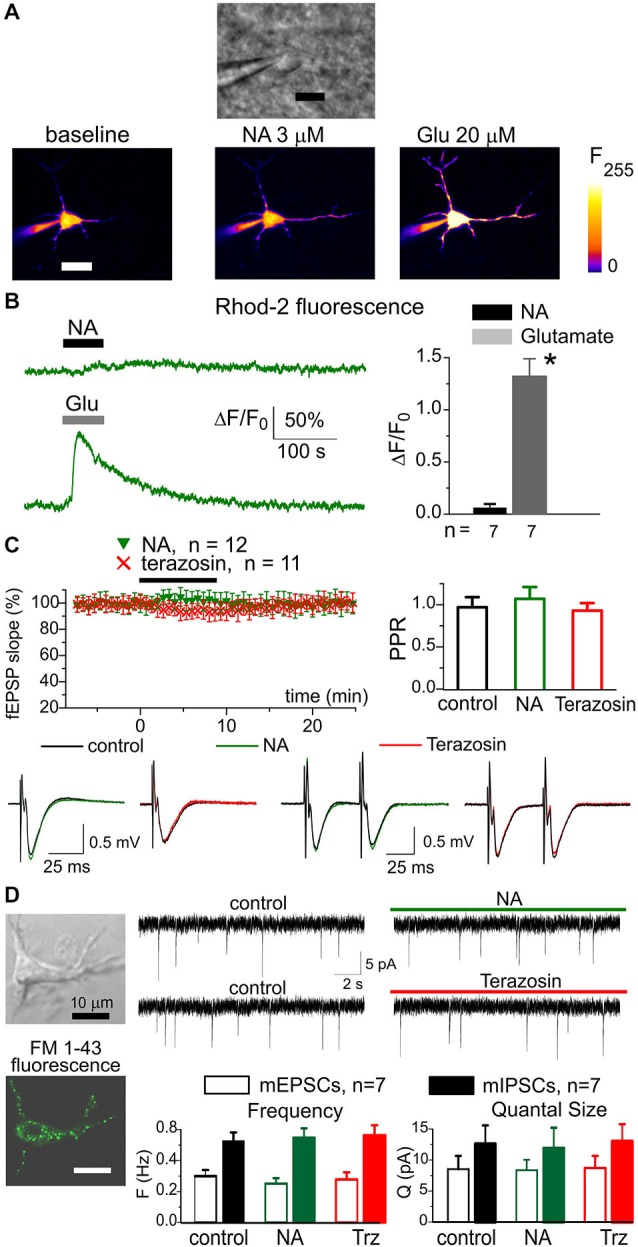
**Noradrenaline did not cause notable response in neocortical neurons**. The pyramidal neurons of somatosensory cortex layer 2/3 of wild-type (WT) mice were loaded with Ca^2+^-indicator Rhod-2 via patch-pipette. Ca^2+^-signals were evoked in neurons by 60 s-long rapid bath application of 3 μM NA and 100 μM L-Glutamate to cortical slices. The membrane holding potential during Ca^2+^-measurements was −40 mV **(A)**, the representative gradient-contrast image and pseudo-color fluorescent images recorded at rest and at the peak of Ca^2+^- responses to NA **(B)**, *left* the representative Ca^2+^-transients evoked in the neuron of dn-SNARE mouse by application of NA and glutamate. *Right*, the diagram shows the pooled data (mean ± SD for number of neurons indicated) of peak Ca^2+^-elevation; the difference between NA and glutamate-evoked response was statistically significant with (*) *P* < 0.005 (paired *t*-test). **(C)** Application of NA and terazosin do not have notable effect on the field excitatory synaptic potentials (fEPSPs) in the neocortical layer 2/3. Upper graphs show time course of fEPSP slope during application of 3 μM NA and 30 nM terazosin and paired pulse ratio in the control and 8 min after the drugs. The graphs below show the representative fEPSPs (average of 10 waveforms). Each dot in the time course shows the average slope of fEPSPs recorded in 1 min time window and normalized to the control; data are presented as mean ± SD for the number of experiments indicated. **(D)** Whole-cell currents were recorded in the isolated neuron retaining functional synaptic boutons. To verify the presence of functional synapses, the cell was pre-incubated with 3 μM FM1–43 for 15 min, then dye was washed out for 15 min. The spontaneous excitatory currents (mEPSCs) were recorded at −80 mV in the presence of picrotoxin (100 μM); the inhibitory mIPSCs were recorded in the presence of NBQX (30 μM). *Left* column: representative fluorescent and gradient contrast image of neuron showing punctate staining with FM1–43. *Right*: the representative mEPSCs before and after application of NA and Terazosin as indicated in the graphs. *Below*, the diagram shows the frequency and quantal amplitude of mEPSCs and mIPSCs pooled for seven neurons. Note that application of NA and terazosin did not change the frequency and amplitude of synaptic currents. These data demonstrate specificity of NA action in the neocortex in respect to the astrocytic and neuronal signaling.

Importantly, we did not observe any significant contribution of α1-AR to neuronal signaling. The amplitude of responses evoked in pyramidal neurons by 3 μM NA was much smaller than the amplitude of glutamate-evoked response (Figures 2A,B). The small NA-evoked response had slower kinetics and started with considerable delay which argues against its origin from direct activation of neuronal α1-ARs. Instead, the neuronal response may have originated from some gliotransmitters released upon activation of glial α1-AR. Furthermore, neither NA nor terazosin exhibited significant effects on fEPSPs in the neocortex (Figure 2C). The application of NA caused a small increase in the slope and paired-pulse ratio of fEPSPs whereas terazosin decreased these parameters. In all cases, the difference from the control was not significant (paired *t*-test, *n* = 12 for NA and 11 for terazosin). To verify the specificity of action of NA and terazosin, we applied these drugs to acutely-isolated neocortical neurons which were devoid of the influence of glial cells (Figure 2D). We used a technique of non-enzymatic vibro-dissociation which allows functional synapses to be maintained on the membrane of isolated neurons, which can be verified by staining with FM1–43 and the presence of miniature spontaneous synaptic currents (Duguid et al., 2007; Rasooli-Nejad et al., 2014). We recorded whole-cell currents in acutely-dissociated neocortical pyramidal neurons at membrane potential of −80 mV. The glutamatergic miniature excitatory postsynaptic currents (mEPSCs) were recorded in the presence of 100 μM picrotoxin and 20 μM PPADS; the GABA-mediated inhibitory currents were recorded in the presence of 30 μM NBQX and 20 μM PPADS. The application of NA and terazosin did not cause notable changes in the amplitude and frequency of mEPSCs and miniature inhibitory postsynaptic currents (mIPSCs; seven isolated neurons were tested in the each case).

Combined, our results show that α1-ARs can make a substantial contribution to Ca^2+^-signaling in neocortical astrocytes. Importantly, our data show that expression of dnSNARE protein in astrocytes did not affect adrenergic signaling.

### Glial Adrenoceptors Induce the Release of ATP and D-serine from Neocortical Astrocytes

**Figure 3:**
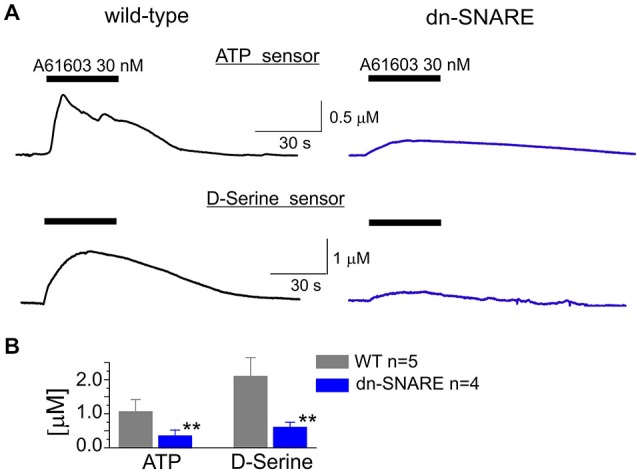
**Adrenoreceptor-activated release of ATP and D-serine in the neocortical slices**. **(A)** The representative responses of cortical slices of WT and dn-SNARE mice to the application of selective α1-AR agonist A61603 (100 nM) were recorded using microelectrode sensors to ATP and D-serine placed in the layer II/III (see Methods). The data are shown as an elevation relative to the resting concentration. **(B)** The pooled data on the peak magnitude of ATP- and D-serine transients evoked by application of A61603; data shown as mean ± SD for number of experiments indicated. Asterisks (**) indicate statistical significance of difference in the magnitude of ATP- and D-serine responses between WT and dn-SNARE mice, *P* < 0.01 (*t*-test). The significant reduction in the NA-evoked responses in the cortical slices from dn-SNARE mice strongly supports the vesicular mechanism of ATP and D-serine release from astrocytes.

To investigate the release of gliotransmitters which could plausibly follow the activation of α1Rs in astrocytes, we used microelectrode biosensors to monitor the concentration of ATP and D-serine in neocortical tissue. This technique was used previously for evaluation of transmitter release in several brain areas (Frenguelli et al., 2007; Rasooli-Nejad et al., 2014). Since NA can be directly oxidized by the sensors and thus generate a measurable sensor current, we used the specific α1-AR agonist A61603. Activation of intracellular Ca^2+^ in astrocytes by A61603 (100 nM) induced a significant increase in the levels of extracellular ATP and D-serine in the cortical tissues of WT mice (Figure 3A).

The α1-AR-mediated elevation of extracellular ATP and D-serine reached 1.1 ± 0.4 μM and 2.1 ± 0.7 μM, respectively (Figure 3). In comparison to the WT littermates, the amplitudes of ATP and D-serine transients were reduced in the dn-SNARE-expressing mice by 69 ± 19% (*n* = 4) and 72 ± 14% (*n* = 4), respectively (Figures 3A,B). This result suggested the astroglial origin and vesicular nature of adrenoceptor-activated release of ATP and D-serine release. It is worth noting that one could not expect a complete inhibition of astroglial exocytosis in the neocortex of dn-SNARE mice since a proportion of astrocytes do not express dn-SNARE (Pascual et al., 2005). Also, there are non-vesicular pathways of gliotransmitter release which might contribute to the adrenoreceptors-triggered response (Hamilton and Attwell, 2010; Montero and Orellana, 2015).

**Figure 4:**
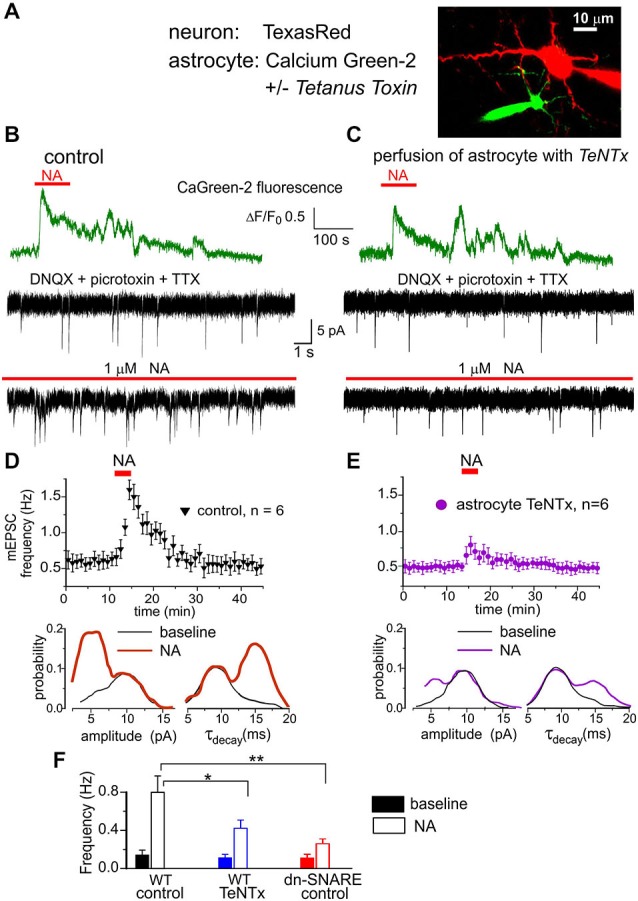
**Astrocyte-derived ATP activates purinergic receptors in the neocortical neurons**. **(A)** Whole-cell currents were recorded in the pyramidal neuron of layer 2/3 simultaneously with perfusion of astrocyte with intracellular solution containing either fluorescent dye Ca-Green-2 alone or Ca-Green-2 and 3 nM of Tetanus neurotoxin light chain. **(B,D)** Activation of astrocytic Ca^2+^ signaling elicited the burst of purinergic currents in the neuron. **(B)** The astrocytic Ca^2+^ transients (green) evoked by application of 1 μM NA and neuronal whole-cell currents (black) recorded in the presence of picrotoxin, DNQX and TTX at −80 mV. Spontaneous currents recorded in these conditions are mediated by the P2X receptors. Upper and lower trace show currents recorded correspondingly before and 1 min after NA application. Note the appearance of the large number of spontaneous currents after NA. **(D)** *Upper graph*: each dot shows the average frequency of spontaneous purinergic currents recorded in 1 min time window in the pyramidal neurons; data are presented as mean ± SD for six neurons. *Lower graphs* show the amplitude and decay time distributions for the purinergic currents recoded before (baseline) and 1–3 min after application of NA. Application of NA elicited the burst of purinergic currents which had slower kinetics and smaller quantal size than currents recorded in the baseline condions. **(C,E)** Perfusion of astrocyte with TeNTx inhibited the burst of purinergic currents verifying their origin from astrocytic exocytosis. **(F)** Diagram shows the frequency of slow purinergic spontaneous currents in the neurons of WT and dn-SNARE mice averaged within 3 min time window before (baseline) and after application of NA in control and during perfusion of astrocytes with TeNTx. Data are shown as mean ± SD for the six neurons. The statistical significance of difference from the WT control values was as indicated (*) *P* < 0.05 and (**) *P* < 0.01 (unpaired *t*-test).

In the next series of experiments we tried to directly verify that astroglial α1-ARs contribute to triggering Ca^2+^-dependent exocytosis. We previously demonstrated that pyramidal neocortical neurons express functional P2X receptors (Pankratov et al., 2007) and these receptors can be activated by ATP released from astrocytes (Lalo et al., 2014a). Thus, pyramidal neurons can be used as a native sensor for extracellular ATP. We recorded whole-cell currents in neocortical pyramidal neurons at a membrane potential of −80 mV in the presence of DNQX (30 μM), D-APV (30 μM) and picrotoxin (100 μM). Similar to our previous experiments (Pankratov et al., 2007; Lalo et al., 2014a), we observed residual non-glutamatergic miniature spontaneous synaptic currents (Figure 4). These non-glutamatergic excitatory spontaneous currents (mEPSCs) were completely abolished by application of specific P2X receptor antagonists PPADS (10 μM) and 5-BDBD (5 μM) in all seven neurons tested (data not shown). Based on these data, as well as our previous work (Pankratov et al., 2007; Lalo et al., 2014a), the phasic inward currents observed in cortical neurons in the presence of glutamatergic and GABAergic antagonists can be confidently attributed to ATP receptors. In order to inhibit astroglial exocytosis, we perfused individual astrocytes with intracellular solution containing 3 nM Tetanus neurotoxin (TeNTx) and 30 μM of the calcium indicator Calcium Green-2 (Figure 4A) and recorded mEPSCs in a neighboring neuron (lying within 30 μm distance from the perfused astrocyte). Perfusion of astrocytes with solution containing only Calcium Green-2 was used as a control. The electrophysiological recordings in neurons started 10–15 min after perfusion of astrocytes with TeNTx.

The purinergic mEPSCs recorded under control conditions had an average amplitude of 8.4 ± 2.5 pA and an average decay time of 9.6 ± 2.6 ms. Application of NA (2 μM) elicited Ca^2+^-elevation in astrocytes and caused a dramatic increase in the frequency of purinergic mEPSCs in all seven neurons tested (Figure 4B). The burst of purinergic currents was accompanied by a decrease in the average amplitude to 6.7 ± 1.7 pA, and an increase in the decay time to 13.3 ± 3.5 ms (*n* = 7). Such behavior was similar to the previously observed burst of purinergic activity activated by astroglial PAR-1 receptors (Lalo et al., 2014a). When astrocytes were perfused with TeNTx, activation of α1-ARs caused much smaller burst of purinergic currents (Figure 4C).

Consistent with our previous reports (Lalo et al., 2014a), purinergic mEPSCs recorded in pyramidal neurons (Figure 4D) exhibited a bimodal amplitude distribution with peaks at 5.8 ± 1.5 pA and 9.8 ± 2.4 pA (*n* = 7). The distributions of mEPSCs decay time in these neurons had peaks at 9.1 ± 1.1 ms and 15.2 ± 2.1 ms. Previously, we demonstrated that purinergic mEPSCs of smaller amplitude and slower kinetics originated from the vesicular release of ATP from astrocytes (Lalo et al., 2014a; Rasooli-Nejad et al., 2014). Application of NA dramatically increased the proportion of these smaller and slower currents (Figure 4E). Perfusion of astrocytes with TeNTx selectively decreased the frequency of slower purinergic mEPSCs, both in control conditions and after application of NA (Figure 4F). These results strongly suggest that smaller and slower purinergic mEPSCs originated directly from vesicular release of ATP from neighboring astrocytes. Incomplete inhibition of the slower mEPSCs can be explained by release from other astrocytes not exposed to TeNTx. NA-evoked bursts of purinergic mEPSCs were significantly inhibited in the dn-SNARE mice, supporting its astroglial origin (Figure 4F).

### Astroglial α1 Adrenoceptors Modulate Long-term Plasticity in the Neocortex

The above data provide strong evidence that astroglial α1-adrenoreceprtors can trigger exocytosis of gliotransmitters, in particular ATP and D-serine (Figures 3, 4). ATP and D-serine have been previously shown to regulate synaptic plasticity in the hippocampus (Pascual et al., 2005; Henneberger et al., 2010) and the neocortex (Lalo et al., 2014b; Rasooli-Nejad et al., 2014). We have shown previously that astrocyte-derived ATP can down-regulate phasic and tonic GABAergic transmission in neocortical neurons. This down-regulation, in synergy with the release of D-serine, can facilitate the induction of LTP (Lalo et al., 2014b; Rasooli-Nejad et al., 2014). Thus, astroglial α1-AR-mediated release of ATP might affect the induction of LTP in neocortical neurons.

**Figure 5:**
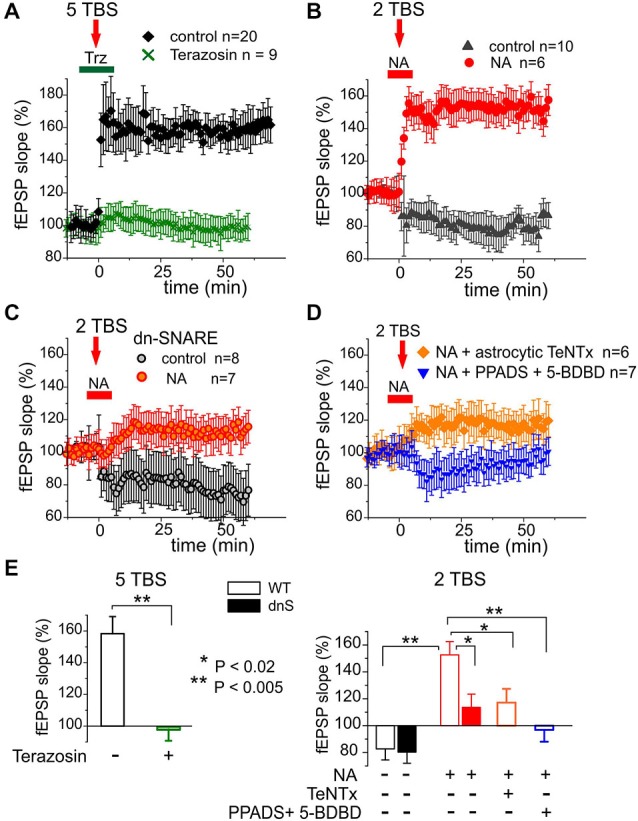
**Release of gliotransmitters and glial α1-ARs are essential for long-term potentiation (LTP) in the neocortex. (A)** LTP was induced in the neocortex of the WT mice by five Θ-bursts of high-frequency stimulation. The magnitude of LTP was significantly reduced by specific antagonist α1-NRs terazosin (30 nM). **(B)** Delivering just two Θ-bursts did not induce LTP in the control but was able to induce LTP when NA (1 μM) was applied during induction as indicated in the graph. **(C)** Application of NA did not facilitate LTP induction in the dn-SNARE mice suggesting the importance of glial exocytosis for the action of NA. **(D)** Effect of NA was decreased by perfusion of astrocyte with TeNTx (similarly to the experiment shown in the Figure 4) or by inhibition of neuronal ATP receptors with PPADS and 5-BDBD. **(E)** Pooled data on the magnitude of LTP evaluated as relative increase in the fEPSP slope at 60th min, averaged across 10 min time window. Each data point shows mean ± SD for the number of experiments indicated in **(A–D)**. Asterisks (*, **) indicate statistical significance of difference in the LTP magnitude WT control values (unpaired *t*-test). These data suggest the importance of NA-triggered release of ATP for glial modulation of LTP in the neocortex.

We investigated the potentiation of fEPSPsin layer II/III of somatosensory cortex of WT and dn-SNARE mice. The fEPSPs were evoked by the stimulation of neuronal afferents descending from layers IV–V. Potentiation of fEPSPs was induced by theta-burst stimulation. In slices from WT mice, five episodes of theta-burst stimulation (5 TBS) induced robust LTP in all 20 experiments (Figure 5A). Application of α1-AR antagonist terazosin inhibited the induction of LTP, suggesting the importance of glial adrenergic signaling. This notion was strongly supported by experiments on the induction of LTP by weaker stimulation (Figures 5B–D). In control conditions, two theta-burst episodes (2 TBS) induced a mild long-term depression rather than potentiation (Figure 4B). The threshold for LTP induction was three theta-bursts (*n* = 12, data not shown). Application of 1 μM NA enabled the induction of LTP with sub-threshold stimulation (2 TBS). Washout of NA 3 min after TBS did not affect the potentiation suggesting the importance of additional NA-mediated activation of astrocytes in the initial period of LTP induction. As mentioned above (Figure 2C), application of NA without TBS did not produce marked potentiation (*n* = 12).

Importantly, the NA-induced facilitation of LTP was significantly reduced in the dn-SNARE mice (Figures 5C,E). In addition, the magnitude of LTP induced by 2 TBS in presence of NA considerably decreased when fEPSPs were recorded in the vicinity of astrocytes perfused with TeNTx (similarly to the experiment with purinergic mEPSCs shown in the Figure 4). The facilitatory effect of NA was abolished by selective inhibition of P2X receptors with 10 μM PPADS and 5 μM 5-BDBD (Figures 5D,E). This result demonstrates the importance of NA-triggered ATP release and activation of ATP receptors for the facilitation of LTP. Combined, our data imply that astroglial α1-ARs contribute to the regulation of exocytosis of gliotransmitters and are important for the synaptic plasticity in the neocortex.

## Discussion

Our data have shown that α1-ARs participate in Ca^2+^-signaling in neocortical astrocytes (Figure 1) but do not have a strong direct action in neurons (Figure 2). We also demonstrate that astrocytic α1-ARs can activate the release of ATP and D-serine from astrocytes (Figure 3). Moreover, we have found that adrenoceptor-activated release of ATP from astrocytes can directly activate postsynaptic P2X receptors in neocortical neurons (Figure 4) and this cascade is involved in astroglial modulation of long-term synaptic plasticity in the neocortex (Figure 5).

### Functional Properties of Astroglial α1-Adrenoreceptors

Our data on the substantial contribution of α1-ARs to Ca^2+^-signaling in neocortical astrocytes agree with previous observations (Ding et al., 2013; Paukert et al., 2014). Moreover, our data reveal specific features of adrenergic signals in cortical astrocytes that have been thus far largely overlooked. Firstly, α1-ARs in cortical astrocytes are very sensitive to NA with an EC_50_ of ~400 nM (Figure 1). Secondly, adrenergic signals in cortical astrocytes undergo amplification by a ryanodine-sensitive Ca^2+^-induced Ca^2+^-release mechanism, in particularly in astrocytic processes (Figure 1A). Nevertheless, application of NA at concentrations greater than 10 μM leads to a long-lasting period of elevated Ca^2+^ and non-responsiveness of astrocytes to subsequent NA (Figure 1B).

The vulnerability of α1AR-mediated responses to desensitization suggests the existence of finely-tuned molecular mechanisms preventing the consequences of their overstimulation, likely to avoid Ca^2+^-overload, which can be very damaging for cells, including astrocytes. Our finding of desensitization of α1-ARs in astrocytes is in line with data obtained previously in other cell types (Akinaga et al., 2013; Jiang et al., 2013). The apparent desensitization of NA-activated responses upon prolonged exposure to an agonist usually originates from their rapid internalization (Mohan et al., 2013; Akinaga et al., 2013). The common molecular mechanisms of adrenoceptor (as well as many other GPCRs) internalization involve beta-arrestins (Mohan et al., 2013) which have been recently implicated in neurodegenerative diseases (Jiang et al., 2013). The, α1-AR-specific internalization cascade involves PKC, which can be activated by elevations in cytosolic Ca^2+^ (Akinaga et al., 2013). Interestingly, the internalization of α1-ARs can also be influenced by their association with the cytoskeleton and lipid rafts (Morris et al., 2008; Akinaga et al., 2013), which can be linked to other astrocytic receptors, for instance P2X1 purinoceptors (Allsopp et al., 2010; Lalo et al., 2011b). The possibility of the activation of purinoceptors by autocrine release of ATP from astrocytes might provide feedback to adrenergic signaling. One might suggest the existence of a variety of mechanisms regulating desensitization and internalization of astrocytic α1-ARs thus underlying an activity-dependent plasticity of astroglial signaling. The study of the molecular mechanisms of desensitization of astrocytic α1-ARa lies beyond the scope of the present work, but this topic surely is of high interest and importance. The plasticity of adrenergic signaling in astrocytes may be very important for glia-neuron interaction and is worth exploring further.

### Astroglial Adrenoreceptors and the Exocytosis of Gliotransmitters

Our data suggest that α1-ARs can trigger the exocytosis of gliotransmitters from neocortical astrocytes (Figures 3, 4). The NA-triggered release of ATP exhibited the same functional properties as release activated by astroglial PAR-1 or CB1 receptors (Lalo et al., 2014a; Rasooli-Nejad et al., 2014). We would like to emphasize that the evidence for a role of vesicular gliotransmitter release in astrocyte-neuron interactions, presented in this study, were obtained mainly using intracellular perfusion of individual astrocytes with Tetanus Toxin light chain (Figures 4, 5). The results obtained by this approach are in a very good agreement with our previous observations of a decrease in the release of ATP and D-serine in dnSNARE mice in which astrocytic vesicular release is compromised (Pascual et al., 2005; Lalo et al., 2014a; Rasooli-Nejad et al., 2014), our data obtained using perfusion of astrocytes with inhibitors of vesicular ATP transporters (Lalo et al., 2014a) and the results obtained using intracellular perfusion with Ca^2+^-chelators (Henneberger et al., 2010). There is also an independent biochemical evidence of storage of D-serine in the synaptic vesicle-like structure in astrocytes (Martineau et al., 2013).

It is worth noting that most of the groups using dn-SNARE mice did not observe any evidence of neuronal expression of the dnSNARE transgene (Pascual et al., 2005; Halassa et al., 2009; Lalo et al., 2014a). Only one study suggested the possibility of neuronal expression of the dnSNARE transgene (Fujita et al., 2014), but it did not provide any evidence for an impairment of neurotransmitter release. Hypothetical neuronal expression of the dn-SNARE transgene should result in a notable deficit in synaptic transmission arising from impaired exocytosis of neurotransmitters. On contrary, there is evidence of up-regulated excitatory (Pascual et al., 2005) and inhibitory (Lalo et al., 2014a) synaptic transmission in the dnSNARE mice. The main deficits that we observed in the dnSNARE mice were in the effects caused by selective activation of Ca^2+^-signaling in the astrocytes via PAR-1 (Lalo et al., 2014a), CB1 (Rasooli-Nejad et al., 2014) or α1-ARs (Figures 3, 4E, 5C). Combined, these results strongly support the physiological importance of vesicular exocytosis from glia and verify the validity of using dn-SNARE mice as a tool to explore astrocyte-neuronal interactions.

The capability of astrocytes to release gliotransmitters is a core element of glia-neuron communication (Halassa and Haydon, 2010; Hamilton and Attwell, 2010). We have shown previously that astrocyte-derived ATP down-regulates inhibitory transmission in pyramidal neurons via phosphorylation of GABA_A_ receptors and thereby can facilitate the induction of long-term synaptic plasticity (LTP) in the neocortex (Lalo et al., 2014b; Rasooli-Nejad et al., 2014). As a source of Ca^2+^-elevation to trigger exocytosis in physiological conditions, our previous data suggested a role for astrocytic NMDA, mGluR and CB1 receptors (Lalo et al., 2014a; Rasooli-Nejad et al., 2014). Our present data highlight the importance of α1-ARs for glial control of synaptic plasticity (Figure 5). The role for adrenergic astroglial signaling may be strengthened by the amplification via ryanodine receptor-mediated Ca^2+^-induced Ca^2+^-release (Figure 1).

### Putative Role for Adrenergic Astroglial Signaling in Metaplasticity

There is growing recognition that information processing in the brain is coordinated by neuronal-glial networks (Halassa and Haydon, 2010; Hulme et al., 2013b; Araque et al., 2014). A key element of this coordination is the ability of astrocytes to integrate neuronal activity over a large spatial domain by virtue of high-affinity receptors (Araque et al., 2014). In comparison to the interaction between neurons, astroglial modulation of synaptic transmission gains many peculiar features, such as slower time-scale, but greater spatial-scale and a dependence upon the combined activity of numerous synapses (Araque et al., 2014). These features can render astrocytes particularly important in heterosynaptic metaplasticity (Panatier et al., 2011; Min and Nevian, 2012; Hulme et al., 2013b).

Contrary to the “classic” release of neurotransmitters into the synaptic cleft, the release of NA occurs from varicosities of adrenergic neurons into brain extracellular fluid, remotely from target cells, so the effective concentration of NA can be rather low. Cortical astrocytes, by virtue of highly-sensitive α1-ARs, are strategically positioned to receive and integrate diffuse adrenergic input from remote adrenergic neurons and pass the information to the local network. Indeed, adrenergic signaling has been shown to modulate the activity of astrocyte networks according to the behavioral state or sensory inputs (Ding et al., 2013; Paukert et al., 2014). Our data suggest that astroglial α1-ARs can affect the induction of long-term changes in synaptic strength (Figure 5). Thus, astroglial adrenoceptors may be of particular importance for brain metaplasticity induced by experience, environmental factors or neurodegenerative disease (Nithianantharajah and Hannan, 2006; Hulme et al., 2013a).

### Specificity of Astroglial Adrenergic Signaling

The ability of adrenoceptors to activate Ca^2+^-signaling in astrocytes encouraged us to use NA for triggering gliotransmission (Gordon et al., 2009; Pougnet et al., 2014). This raises a question as to whether the action of NA is astroglia-specific. Our data show that principal neocortical neurons do not produce Ca^2+^-transients in response to application of NA (Figures 2A,B). The application of NA produced a small effect on excitatory synaptic potentials (Figure 2C), which could possibly be attributed to the modulatory action of gliotransmitters. This notion is strongly supported by the lack of any notable effect of NA on synaptic currents in acutely-isolated neurons that are devoid of astrocyte influence (Figure 2D). In contrast, NA-elicited phasic purinergic currents and NA-elicited enhancement of LTP were significantly reduced by selective inhibition of astrocytic exocytosis (Figures 4, 5). Thus, the observed effects of NA and terazosin on LTP can hardly be related to neuronal adrenoreceptors. Our data strongly suggest that astrocytes provide a major contribution to adrenergic modulation of synaptic transmission and plasticity in the neocortex.

Other types of glial cells, in particular microglia, can also express adrenoceptors (Gyoneva and Traynelis, 2013; Butt et al., 2014). Microglia and oligodendocytes can also release ATP and other gliotransmitters molecules, but mainly via hemichannels rather than exocytosis (Butt, 2011; Montero and Orellana, 2015). Most of the observations of microglial release of ATP were made in a pathological context, e.g., neuroinflammation or ischemia (Butt, 2011; Montero and Orellana, 2015). Hemichannel-mediated pathways of ATP release do not agree with the fast kinetics and quantal behavior of the NA-elicited purinegic currents we observe in neurons (Figure 4). Furthermore, microglial cells were reported to express β2-rather than α1-adrenoceptors (Gyoneva and Traynelis, 2013; Butt et al., 2014). This argues against their significant contribution to the regulation of neocortical LTP which is sensitive to the α1-AR antagonist terazosin (Figure 5). Still, one cannot *a priori* expect absolute astroglial selectivity of NA’s action: the role for microglia and oligodendrocytes in adrenergic modulation of synaptic transmission are yet to be established.

Our observation of a lack of direct NA action on synaptic potentials contrasts with the results obtained in the hippocampus, where NA caused a depression of fEPSPs and accelerated the hypoxic depression of synaptic transmission (Pearson and Frenguelli, 2004). Most likely, the adrenergic modulation of synaptic transmission in the hippocampus occurred via α2-AR-mediated presynaptic inhibition and β1-AR- activated increase in extracellular adenosine (Pearson and Frenguelli, 2004). It remains unclear whether glial adrenoreceptors can contribute to these mechanisms, e.g., by activating the release of ATP/adenosine. It is worth noting that recent work by Pougnet et al. (2014) also reported NA-induced synaptic depression in hippocampal neurons but suggested a different mechanism, involving α1-AR-activated release of ATP from astrocytes and activation of postsynaptic purinoreceptors causing Ca^2+^-dependent internalization of AMPA receptors. Thus, adrenergic modulation of synaptic transmission can involve multiple mechanisms and show significant regional differences. In each particular case, special care is needed to distinguish the putative involvement of glial and post- and presynaptic neuronal adrenoreceptors.

To conclude, our results strongly support the physiological importance of astroglial adrenergic signaling and the astrocytic exocytosis of gliotransmitters. This adrenoreceptor-mediated communication between astrocytes and neurons is necessary for the regulation of synaptic strength and the modulation of synaptic plasticity.

## Conflict of Interest Statement

The authors declare that the research was conducted in the absence of any commercial or financial relationships that could be construed as a potential conflict of interest.
